# Aggressive FLC Escape in a Patient with IgD Myeloma

**DOI:** 10.1155/2015/694730

**Published:** 2015-11-10

**Authors:** Cédric Farges, Murielle Roussel, Anne Huynh, Antoine Blancher, Bénédicte Puissant-Lubrano

**Affiliations:** ^1^Laboratoire d'Immunologie, CHU de Toulouse, Hôpital Rangueil, 1 Avenue Jean Poulhès, TSA 50032, 31 059 Toulouse Cedex 9, France; ^2^Hematology Department, University Hospital, 31059 Toulouse, France; ^3^Laboratoire d'Immunogénétique Moléculaire (EA3034), Faculté de Médecine Toulouse-Rangueil, Université Paul Sabatier Toulouse III, Bâtiment A2, 133 route de Narbonne, 31062 Toulouse Cedex 04, France

## Abstract

*Background*. Some patients who are stable or in remission from a myeloma secreting intact monoclonal immunoglobulin (+/− associated free light-chains (FLCs)) relapse with production of FLC. This FLC escape is one of the illustrations of the intraclonal heterogeneity of multiple myeloma.* Results*. We report FLC escape in a patient with IgD myeloma characterized by a severe outcome. We discuss parameters that negatively impacted prognosis in this patient, including bone lesions, biochemical parameters, and genomic abnormalities.* Conclusion*. This case illustrates the selective pressure exerted by therapeutic drugs and the variable sensitivity of subclones to these drugs; it also highlights the importance of FLC monitoring in treated MM patients.

## 1. Introduction

Free light-chains (FLCs) escape is a pattern of relapse observed in 2.5 to 8% of treated IgG and IgA multiple myeloma (MM) patients [1, 2]. It concerns patients who presented with an intact monoclonal immunoglobulin, alone or associated with FLC, and who relapsed after treatment with raising production of FLC associated with stable or decreased intact Ig. In case of patients who presented with intact immunoglobulin associated with FLC (who represent almost 90% patients with intact immunoglobulin), the relapse appeared as an increase of FLC without a parallel rise of the intact immunoglobulin [1–3]. FLC escape illustrates the intraclonal heterogeneity of multiple myeloma and was recently associated with shortened overall survival [2]. We describe the first case of FLC escape in an IgD MM patient. This case was very aggressive and we will discuss parameters that negatively impacted prognosis in this patient.

## 2. Case Presentation

A 50-year-old man presented in 2008 with a backache. X-rays and MRI showed multiple lytic bone lesions of the spine and pelvis. He also had renal involvement and anemia while calcemia and LDH were within the reference ranges (see Table 1).

Serum electrophoresis revealed a monoclonal protein, which was identified by immunofixation electrophoresis (IFE) as IgD lambda associated with monoclonal lambda FLC. IgD was quantified at 7600 mg/L (radial immunodiffusion, Bindarid IgD, The Binding Site) and FLCs were measured at 3590 mg/L (Freelite assay, Immunonephelometer Immage 800, Beckman Coulter). Urinary IFE revealed monoclonal lambda FLCs. A bone marrow aspirate showed the presence of 35% dysmorphic plasma cells. Therefore the diagnosis of IgD multiple myeloma (MM) was made (ISS stage III). FISH analysis did not reveal 13q14 nor 17p deletions. The search for translocation t(4;14) was inconclusive.

The patient was enrolled in the clinical trial IFM 2007-02 (NCT00910897). A 4-cycle treatment associating Bortezomib and Dexamethasone was initiated followed by high dose therapy with Melphalan 200 mg/m^2^ followed by autologous stem cell transplantation in February 2009.

**Figure 1:**
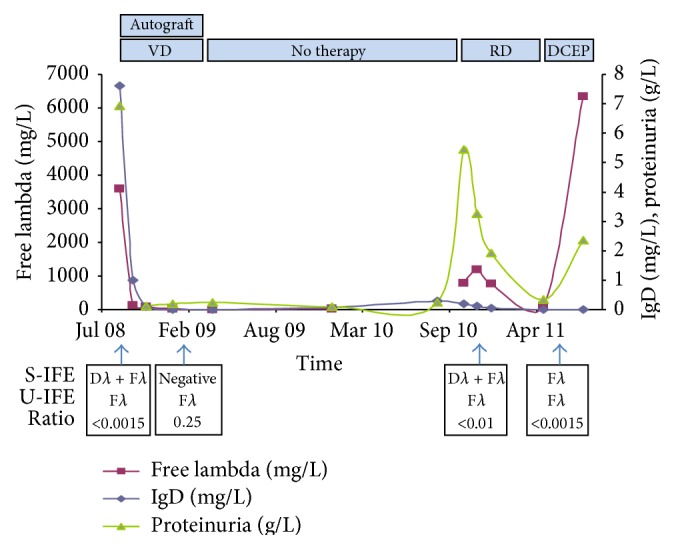
Graph shows the evolution of IgD, lambda-free light-chains, and proteinuria. The different treatments appear at the top (blue rectangles) and the results of IFE below the graph. S-IFE: serum immunofixation electrophoresis, U-IFE: urinary immunofixation electrophoresis, VD: velcade dexamethasone, RD: revlimid dexamethasone, and DCEP: dexamethasone cyclophosphamide etoposide cisplatin.

The patient achieved a very good partial response (VGPR) with a serum M-protein undetectable on IFE but monoclonal lambda FLCs were still detectable on urinary IFE. During this period, serum lambda FLC and IgD were within the normal range (Table 1 and Figure 1).

The patient relapsed in December 2010 as he presented with a skull vault tumor. IgD was quantified at 204 mg/L and FLCs were measured at 779 mg/L. Patient started on radiotherapy plus high dose Dexamethasone. In May 2011, while on therapy, the patient presented with epiduritis and spinal cord compression. IFE identified monoclonal lambda FLC, which was quantified at 6330 mg/L (Table 1). A treatment associating methylprednisolone, radiotherapy followed by 3 cycles of dexamethasone cyclophosphamide etoposide cisplatin was initiated. The patient died in August 2011 of MM progression.

## 3. Discussion

IgD MM is a rare entity representing 1-2% of MM. IgD MM patients are more likely to present signs of aggressive disease at diagnosis and, accordingly, IgD MMs have been associated with shorter survival and reduced response to treatment than IgG and IgA MM [4, 5]. Our patient displayed signs of high-risk features at diagnosis such as advanced ISS stage (III), high rate of bone lesions (*N* = 3), anemia, and elevated levels of both *β*2-microglobulin (>5.5 mg/dL) and creatinine. He also displayed clinical/biochemical features that are common to IgD MM, that is, a young age (<65 years), a male gender, absence of extramedullary plasmacytoma, and a lambda light-chain [6, 7]. However, the worse outcome of IgD MM can also be linked to a delayed diagnosis of IgD myeloma because it requires IFE with anti-IgD antibody which is not performed in first line [8]. In addition, the use of autologous stem cell transplantation and high dose chemotherapy including “recent” drugs (proteasome inhibitors) has recently improved the outcome of patients suffering from IgD myeloma [7, 9]. A long-term complete response has been described in IgD myeloma patient [10]. Therefore, although recent studies have highlighted some differences between IgD MM and other Ig MM subtypes [6, 11], a worse outcome cannot be considered anymore as an inherent characteristic of IgD MM [9].

FLC escape is one of the illustrations of the intraclonal heterogeneity of multiple myeloma [1, 2]. Heterogeneity of plasma cells in MM has been recently documented by molecular biology studies on sequential samples from MM patients [12–14] and was also demonstrated at the stage of MGUS [14]. These studies reported three evolution types at the molecular level in MM patients, each of them concerning approximately one-third of patients: (i) no modification between diagnosis and relapse, (ii) evolution of the major subclone at diagnosis through acquisition of genetic lesions, and (iii) expansion of a minor clone at diagnosis [12, 13]. The latter evolution called nonlinear evolution has been observed preferentially in patients treated with Bortezomib/Dexamethasone and after a complete response or a VGPR [12]. Our patient perfectly illustrates this evolution pattern as he relapsed with lambda FLC after presentation with intact IgD and after a VGPR following Bortezomib and Dexamethasone. In our case, the MM heterogeneity was biochemically detectable by IFE and FLC quantification.

Clonal heterogeneity in MM patients has been associated with the presence of high-risk genomic abnormalities such as t(4;14); t(14;16); t(14;20); or del17p13 [13]. Our patient did not have 13q14 or 17p deletions. Unfortunately, the search for translocation t(4;14) was inconclusive. Of note, he had a high level of *β*2-microglobulin, which was previously reported to be an independent predictor of poor survival [15].

In accordance with the association of clonal heterogeneity and high-risk genomic abnormalities, FLC escape has been associated with a worse evolution than relapse with an intact immunoglobulin [2]. Our patient had an overall survival (OS) of 36 months after a very good partial response to first line chemotherapy with Bortezomib/Dexamethasone and AHSCT. This OS is in agreement with the median OS of IgD MM patients after autologous SCT (30 months [6]) but is lower than the median OS reported for patients with FLC escape after IgG or IgA MM (47.5 months [2]). However, our patient‘s survival from FLC escape was only three months, while the median survival from FLC escape relapse was 27.7 months in IgG and IgA MM patients [2], illustrating a very aggressive relapse as FLC escape.

The case presented here illustrates the clonal heterogeneity in a patient with IgD MM associated with a severe outcome. This FLC escape in an IgD MM is the first documented in English language to our knowledge. The clonal heterogeneity of MM revealed by a different clone at diagnosis and relapse illustrates the selective pressure exerted by therapeutic drugs and the variable sensitivity of subclones to these drugs [12], even if we cannot rule out an evolution reflecting the natural history of the disease and the competition between subclones. The studies cited above have clinical implications and highlight the necessity to adapt treatment to this heterogeneity in order not to favour the emergence of resistant subclones.

Secondly, this case confirms earlier observations on the importance of FLC monitoring in treated MM patients. This also includes MM patients without FLC at diagnosis because it was reported that 11% of FLC escape occurred in patients with intact Ig and without FLC at diagnosis (iFLC < 100 mg/L) [2]. In our case, the biochemical relapse objectified by IFE and FLC assay did not precede the clinical relapse because our patient lived in Africa without a regular follow-up. However, FLC assay is the more sensitive and reliable assay routinely available for FLC assessment compared to electrophoresis and IFE [16] and is also more accessible and widespread than molecular biology.

## Figures and Tables

**Table 1:** Biochemical characteristics of the patient.

	Diagnosis	VGPR	Relapse	FLC escape
	August 2008	2009	December 2010	May 2011
M-protein at electrophoresis (g/L)	4.4	No	0.35	1.30
Serum IFE	IgD *λ* + free lambda	Negative	IgD *λ* + free lambda	Free lambda
Urinary IFE	Free lambda	Free lambda	Free lambda	Free lambda
IgD (mg/L)	7600	<90^*∗*^	204	<8.5
Lambda free light-chain (mg/L)	3590	<23^*∗*^	779	6330
Proteinuria (g/L)	6.92	<0.25^*∗*^	5.45	2.36
Creatinine (*µ*mol/L)	139	<107^*∗*^	116	303
Hemoglobin (g/dL)	9	12.8	10.3	9.0
Calcemia (mmol/L)	2.49	2.26	2.47	2.41
LDH (UI/mL)	344	<488	689	944

Reference values:

IgD 1.3–152.7 mg/L; lambda free 5.7–26.3 mg/L; proteinuria >0.1 g/L; creatinine 64–104 *µ*mol/L; hemoglobin 13–17.5 g/dL; calcemia 2.20–2.60 mmol/L; LDH 210–450 UI/mL.

^*∗*^The maximal value observed during the remission is presented.

## Abbreviations

FLC: Free light-chain
IFE: Immunofixation electrophoresis
MM: Multiple myeloma
VGPR: Very good partial response.
